# Pubertal attainment and Leydig cell function following pediatric hematopoietic stem cell transplantation: a three-decade longitudinal assessment

**DOI:** 10.3389/fendo.2023.1292683

**Published:** 2023-12-13

**Authors:** Alessandro Cattoni, Maria Laura Nicolosi, Giulia Capitoli, Alberto Gadda, Silvia Molinari, Sotiris Louka, Andrea Buonsante, Simona Orlandi, Gianluca Salierno, Iacopo Bellani, Francesca Vendemini, Giorgio Ottaviano, Alberto Gaiero, Graziella Fichera, Andrea Biondi, Adriana Balduzzi

**Affiliations:** ^1^ Department of Pediatrics, Fondazione IRCCS San Gerardo dei Tintori, Monza, Italy; ^2^ Department of Medicine and Surgery, University of Milano-Bicocca, Monza, Italy; ^3^ Bicocca Bioinformatics, Biostatistics and Bioimaging B4 Centre, University of Milano-Bicocca, Monza, Italy; ^4^ Department of Pediatrics and Neonatology, IRCCS Gaslini Savona e Pietra Ligure, Savona, Italy; ^5^ Department of Endocrinology, Ospedale San Paolo, Savona, Italy

**Keywords:** treosulfan, busulfan, total body irradiation, hypogonadism, gonadal disorders, hematopoietic stem cell transplantation, testosterone

## Abstract

**Introduction:**

Impaired testosterone secretion is a frequent *sequela* following hematopoietic stem cell transplantation (HSCT) in pediatrics, but long-term longitudinal trendlines of clinical and biochemical findings are still scanty.

**Methods:**

Monocentric, retrospective analysis. Male patients transplanted <18 years between 1992 and 2021, surviving ≥2 years after HSCT and showing, upon enrollment, clinical and biochemical signs consistent with pubertal onset and progression were included. Clinical and biochemical data collected every 6-12 months were recorded.

**Results:**

Of 130 patients enrolled, 56% were prepubertal, while 44% were peri-/postpubertal upon HSCT. Overall, 44% showed spontaneous progression into puberty and normal gonadal profile, while the remaining experienced pubertal arrest (1%), isolated increase of FSH (19%), compensated (23%) or overt (13%) hypergonadotropic hypogonadism. Post-pubertal testicular volume (TV) was statistically smaller among patients still pre-pubertal upon HSCT (*p* 0.049), whereas no differences were recorded in adult testosterone levels. LH and testosterone levels showed a specular trend between 20 and 30 years, as a progressive decrease in sexual steroids was associated with a compensatory increase of the luteinizing hormone. A variable degree of gonadal dysfunction was reported in 85%, 51%, 32% and 0% of patients following total body irradiation- (TBI), busulfan-, cyclophosphamide- and treosulfan-based regimens, respectively. TBI and busulfan cohorts were associated with the lowest probability of gonadal event-free course (*p*<0.0001), while it achieved 100% following treosulfan. A statistically greater gonadotoxicity was detected after busulfan than treosulfan (*p* 0.024). Chemo-only regimens were associated with statistically larger TV (*p <*0.001), higher testosterone (*p* 0.008) and lower gonadotropin levels (*p <*0.001) than TBI. Accordingly, the latter was associated with a 2-fold increase in the risk of gonadal failure compared to busulfan (OR 2.34, CI 1.08-8.40), whereas being pre-pubertal upon HSCT was associated with a reduced risk (OR 0.15, CI 0.08-0.30).

**Conclusions:**

a) patients pre-pubertal upon HSCT showed a reduced risk of testicular endocrine dysfunction, despite smaller adult TV; b) patients showed downwards trend in testosterone levels after full pubertal attainment, despite a compensatory increase in LH; c) treosulfan was associated to a statistically lower occurrence of hypogonadism than busulfan, with a trend towards larger TV, higher testosterone levels and lower gonadotropins.

## 1 Introduction

Hematopoietic stem cell transplantation (HSCT) has been historically regarded as a pivotal curative option for children and adolescents with high-risk, relapsed or refractory hematological malignancies (1, 2). In this setting, the refinement of supportive therapies, the development of patient-tailored targeted treatments and the enhancements in donor-selection procedures has resulted in outstanding improvements in the post-transplantation survival rates over the last decades (3).

In addition, HSCT has become the standard-of-care for a growing body of non-hematological and non-malignant disorders, such as hemoglobinopathies or inborn error of metabolism or immunity (4–7).

The dramatical increase in the worldwide population of survivors exposed to HSCT during childhood, adolescence or young adulthood has progressively shed light on the burden of adverse events experienced years or even decades following transplantation, as a result of the synergetic detrimental action of irradiation, alkylating agents, graft versus host disease (GvHD) and eventual front-line antineoplastic treatments. Accordingly, the scientific community has witnessed the onset of a new medical scenario focused on monitoring, timely diagnosis and early treatment of late adverse events following HSCT.

Endocrinopathies represent a remarkable share of all the medical sequelae after transplantation in pediatrics, with variable degrees of gonadal and thyroid impairment being reported in almost 60% of the patients transplanted before the age of 10 years (8), but with a wide variability related to the conditioning regimen administered.

The term “conditioning” indicates the preparative treatment delivered prior to HSCT with the purpose of reducing the tumor burden, in case of a malignant disorder, and allowing donor stem cell engraftment, whichever the disease. Based on clinical indication and host-related features, the conditioning regimen consists of either chemotherapy alone or a combination of chemo- and radiotherapy (total body irradiation, TBI).

Both irradiation and alkylating agents severely affect testicular function. Sertoli cells are remarkably more sensitive to the detrimental action of chemotherapy and radiotherapy than testosterone-secreting Leydig cells (9). Accordingly, the reported prevalence rates of azoospermia and infertility achieve 85% in mixed cohorts of patients transplanted for either malignant or non-malignant disorders and a higher proportion in patients transplanted for acute lymphoblastic leukemia after a TBI-based regimen (10). On the other hand, the occurrence of testosterone deficiency is remarkably lower, ranging from 0% following the exposure to chemo-only regimens to 56% among patients exposed to TBI (11).

While the deleterious action of irradiation on testicular endocrine function has been already extensively investigated, conflicting results have been reported about radiation-free conditioning schedules. In detail, busulfan has been historically regarded as the most gonadotoxic alkylating agent in both genders (12), while a growing body of literature has more recently highlighted a gonadal-sparing profile of treosulfan in animal models and in female patients (13, 14). Though a trend towards milder gonadotoxicity of treosulfan has been depicted also among male transplanted patients, no statistical significance has been achieved by published analyses, to date (15).

In addition, from a biological perspective, we are now aware that iatrogenic gonadal damage is a dynamic phenomenon, as radiotherapy and alkylating agents anticipate at a variable pace the physiological decrease in residual endocrine activity commonly experienced later in life in unaffected individuals from both genders. Nevertheless, most authors provide cross-sectional evaluations of gonadal outcomes following HSCT, without describing the time-dependent changes in testicular function over time.

In order to fill these gaps of knowledge, we conducted the present analysis and reported the longitudinal trendlines of clinical and biochemical markers of Leydig cell function over a dedicated three-decade endocrine follow-up in a monocentric cohort of male survivors after pediatric HSCT. In addition, we aimed at characterizing the specificities of the long-standing effects of different conditioning regimens on testosterone secretion.

## 2 Materials and methods

### 2.1 Study design and patients’ enrollment

All male patients alive more than 24 months following HSCT and who were transplanted before the age of 18 years between 1st January 1992 and 31st January 2021 at the Hematopoietic Stem Cell Transplant Unit of Fondazione IRCCS San Gerardo dei Tintori in Monza were potentially eligible for this retrospective, monocentric, observational study. All patients were offered a longitudinal endocrine long-term follow-up program and to be periodically evaluated by pediatric endocrinologists with a commitment in the field of post-HSCT late effects. Patients who adhered to the program with three or more endocrine evaluations and for whom either clinical and/or biochemical signs consistent with spontaneous onset of puberty (Tanner stage ≥2 and/or testicular volume ≥ 4 mL) were documented or for whom pharmacological pubertal induction was undertaken due to pubertal delay were included in the study population.

The following exclusion criteria were considered: mono- or bilateral orchidectomy; 2 Gy-TBI undertaken as a part of the conditioning regimens for severe aplastic anemia; patients exposed to testicular/inguinal/pelvic irradiation prior to the conditioning, as a part of front-line treatment; CNS tumors involving the hypothalamic pituitary area and/or exposure to cranial irradiation; prolactin-secreting tumors; congenital mono- or bilateral cryptorchidism.

All the patients involved in the study provided written informed consent to data collection and analysis in an international Registry, within the umbrella of the European Bone and Marrow Transplantation Society, and to their dissemination in the setting of local, National or International studies. The Registry has been approved by the local Ethical Committee (Comitato Etico della Brianza). The aim of the Registry is to provide a pool of data and facilitate studies investigating several transplantation-related issues for scientific purposes, including endocrine iatrogenic disorders.

### 2.2 Data collection

The following variables have been collected: a) data on background disease: diagnosis, age at onset, eventual antineoplastic therapy administered before HSCT, cumulative dose of alkylating agents administered, complete remission achieved before HSCT, if applicable; b) features of HSCT: age upon transplantation, conditioning regimen, donor compatibility, overall duration of immunosuppression, cell source, maximum acute and/or chronic graft versus host disease (GvHD) grading/scoring; c) clinical data recorded upon periodic endocrine assessment: Tanner stage, testicular volume (assessed by Prader’s orchidometer), degree of virilization; d) laboratory data recorded longitudinally every 6 to 12 months: LH, FSH, total testosterone, glycaemia, glycated hemoglobin, insulin, total cholesterol, HDL, LDL and triglyceride levels; e) bone mineral density recorded upon longitudinal DEXA scan evaluations.

The abovementioned demographic, clinical, and laboratory variables were retrieved from medical records, institutional databases, and laboratory and radiology softwares.

In order to assess the potential detrimental contribution of chemotherapy on gonadal health, we estimated for each patient the overall dose of alkylating agents administered before HSCT, either as front-line treatment or as a part of the conditioning regimen. As patients receive polychemotherapy and are therefore exposed to several antineoplastic agents, the dose of each alkylating medication was normalized into the corresponding dose of cyclophosphamide (CED - cyclophosphamide equivalent dose) with a dedicated algorithm: CED (mg/m2) = 1.0 [cumulative cyclophosphamide dose (mg/m2)] + 0.244 [cumulative ifosphamide dose (mg/m2)]+0.857 [cumulative procarbazine dose (mg/m2)]+ 14.286 [cumulative chlorambucil dose (mg/m2)]+15.0 [cumulative carmustine dose (mg/m2)]+ 16.0 [cumulative lomustine dose (mg/m2)] + 40 [cumulative melphalan dose (mg/m2)]+50 [cumulative thiotepa dose (mg/m2)]+100 [cumulative chlormethine dose (mg/m2)]+8.823 [cumulative busulfan dose (mg/m2)] (12). Accordingly, CED was assessed to quantify the overall exposure to gonadotoxic treatments and to compare different antineoplastic protocols.

As a conversion factor from treosulfan to cyclophosphamide has not been published yet, CED was not available for patients conditioned with this alkylating agent.

### 2.3 Definitions and classifications

Based on the *pubertal status* recorded upon HSCT, the whole population has been subdivided into two study cohorts: the pre-pubertal (PreP) cohort, that included patients with neither clinical nor laboratory signs consistent with pubertal onset upon HSCT versus the peri-/post-pubertal (PostP) cohort, that included either boys for whom puberty was already kicking-in upon HSCT or patients who had already achieved full pubertal attainment upon transplantation.

In order to assess the detrimental effect played by different *conditioning regimens*, all patients were grouped in the following classes, based on the conditioning administered at HSCT: a) TBI-based; b) Busulfan-based; c) Treosulfan-based; d) other chemo-only regimens, that included all those patients exposed to none of the abovementioned alkylating agents.

In terms of the *gonadal outcomes* found among survivors, the residual testicular function recorded for each patient at the end of a long-lasting endocrine follow-up was defined as follows, based on the latest published guidelines about testosterone deficiency (16–18): a) Normal testicular endocrine function (NOR): patients who achieved full pubertal development, LH < 10 U/L, FSH < 15 U/L, testosterone ≥ 3 ng/mL and no signs/symptoms consistent with hypogonadism; b) pubertal delay/arrest (PA): lack of progression of Tanner stage for 12 sequential months or more along with progressive increase in LH/FSH and decrease in testosterone levels, prompting the prescription of hormonal therapy to induce pubertal attainment; c) isolated increase of FSH (IIF): LH < 10 U/L, FSH ≥ 15 U/L, testosterone ≥ 3 ng/mL and no signs consistent with hypogonadism; d) compensated hypergonadotropic hypogonadism (cHH): Tanner 5 patients who spontaneously achieved complete pubertal attainment, with raised gonadotropins (LH ≥ 10 U/L, FSH ≥ 15 U/L) but testosterone levels persistently ≥ 3 ng/mL and no symptoms consistent with hypogonadism; e) overt hypergonadotropic hypogonadism (oHH): Tanner stage 5 patients who spontaneously achieved complete pubertal attainment, but showing raised gonadotropins in the setting of either testosterone levels persistently < 3 ng/mL or signs/symptoms consistent with hypogonadism *plus* testosterone persistently <3.5 ng/mL. See **Table 1** for a schematic summary of each definition.

**Table 1:** Definition and classification of the gonadal findings following HSCT in the study population.

Gonadal outcomes	LH (U/L)	FSH (U/L)	Testosterone (ng/ml)	Pubertal attainment and signs/symptoms of hypogonadism
**Normal testicular endocrine function (NOR)**	<10	<15	Post-pubertal: ≥3	Tanner 5 achieved spontaneously – No signs/symptoms of hypogonadism in adulthood
**Pubertal arrest (PA)**	≥10	≥15	Inadequate increase of testosterone levels over puberty	Pharmacological induction of puberty needed to achieve Tanner 5 stage
**Isolated increase of FSH (IIF)**	<10	≥15	≥3	Tanner 5 achieved spontaneously – No signs/symptoms of hypogonadism
**Compensated hypergonadotropic hypogonadism (cHH)**	≥10	≥15	Post-pubertal: ≥3	Tanner 5 achieved spontaneously – No signs/symptoms of hypogonadism in adulthood
**Overt hypergonadotropic hypogonadism (oHH)**	≥10	≥15	<3 in case of asymptomatic patients *or* < 3.5 in case of symptomatic patients	Tanner 5 achieved spontaneously – Signs/symptoms of hypogonadism eventually reported by the patient

LH, FSH and testosterone levels were all assessed by Electrochemiluminescence - ECLIA Elecsys Immunoassay (Roche Diagnostics, Mannheim, Germany). Over the long-term observation period, the immunoassay was upgraded several times. This resulted in an increase in the functional sensitivity of the test, though it did not lead to any clinically remarkable inter-assay variability.

### 2.4 Statistics

Continuous variables were described with mean (SD) and categorical variables were reported as count and frequency. The chi-square test and t-test were used to make comparisons between groups in terms of categorical and continuous variables, respectively.

The temporal trends of testicular growth, testosterone and LH values among patients from the PreP versus the PostP cohorts were estimated by a nonparametric regression model using cubic splines.

Event-free survival was defined as the time from the date of HSCT to the first adverse event or last follow-up (censored observation), whichever occurred first. In this setting, adverse events were defined as either PA, IIF, cHH or oHH, while a retained endocrine function encompassed patients from the NOR cohort.

Survival curves were estimated with the Kaplan–Meier method, whereas the log-rank test was applied to compare the survival curves of different groups (i.e., subcohorts of patients exposed to different conditioning regimens).

A Cox proportional-hazards regression model was used to investigate the association between conditioning regiment, background disease, chronic GvHD, pubertal stage upon HSCT, age upon the last biochemical evaluation and gonadal impairment. Results of the Cox models are expressed in terms of estimated hazard ratios (HR), 95% confidence intervals (95% CI) and *p* values.

The tests performed were 2-sided, and the significance level was set as *p* < 0.05. All statistical analyses were performed using open-source R software v.4.4.2 (R Foundation for Statistical Computing, Vienna, Austria).

## 3 Results

### 3.1 Study population

**Figure 1:**
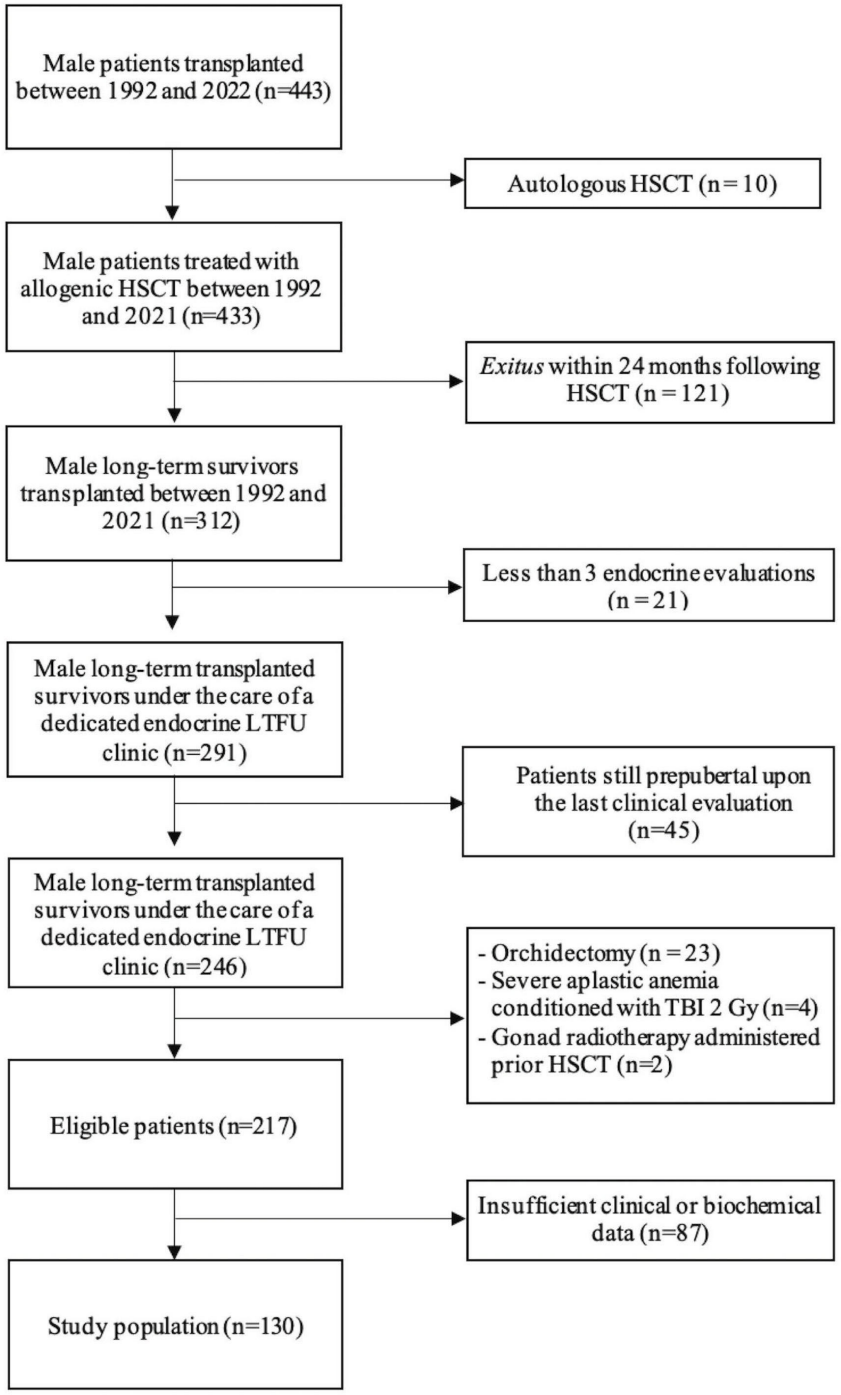
Stepwise selection process that led to the identification of the study population.

Out of 443 male patients transplanted at our Institution between 1992 and 2021, 130 fulfilled enrollment criteria and were included in the study population, as depicted in **Figure 1**.

**Table 2** shows the distribution of the enrolled patients by background disease and conditioning regimen. Overall, 63 patients (48.5%) were transplanted for acute lymphoblastic leukemia (ALL), 21 (16.2%) for acute myeloid leukaemia (AML), 13 (10.0%) for bone marrow failure, 8 (6.2%) for Hodgkin or non-Hodgkin lymphomas (HL/NHL), 8 (3.1%) for other malignant hematological disorders, 6 (4.6%) for hemoglobinopathies, 5 (3.8%) for hemophagocytic lymphohistiocytosis (HLH), 4 (3.1%) for inborn errors of metabolism and 2 (1.5%) for primary immunodeficiencies (PID).

**Table 2:** Background disease and conditioning regimens for the 130 patients enrolled in the study population, displayed by decreasing occurrence.

Disease class	Disease	Conditioning class	Conditioning regimens	Number of patients			
**Acute leukemias**	Acute lymphoblastic leukamiea (ALL)	Busulfan-based	Bu, Cy, Mel	2	7	63	84 (64.6%)
			Bu, Cy, Eto	1			
			Bu, Flu, TT	4			
		TBI-based	TBI, Eto	44	53		
			TBI, Eto, Cy	9			
		Treo-based	Treo, Flu		3		
	Acute myeloid leukaemia (AML)	Busulfan-based	Bu, Cy, Mel	17	18	21	
			Bu, Cy, TT	1			
		Treo-based	Treo, TT, Flu		2		
		Other chemo-only	Flu, Mel		1		
**Bone marrow failures**	Severe aplastic anemia (SAA)	Treo-based	Treo, Flu		1	11	13 (10%)
		Other chemo-only	Cy	3	10		
			Cy, Flu	7			
	Fanconi Anemia (FA)	Other chemo-only	Cy, Flu			2	
**Lymphomas**	Hodgkin Lymohoma (HL)	Other chemo-only	Cy, Flu		1	3	8 (6.2%)
			Flu, Mel		2		
	Non-Hodgkin Lymphoma (NHL)	TBI-based	TBI, Eto	3	4	5	
			TBI, Eto, Cy	1			
		Treo-based	Treo, TT, Flu		1		
**Other malignant disorders**	Chronic myeloid leukaemia (CML)	Busulfan-based	Bu, Cy, Mel		2	4	8 (6.2%)
		Other chemo-only	Flu, Mel, TT		2		
	Juvenile myelomonocytic leukaemia (JMML)	Busulfan-based	Bu, Cy, Mel			1	
	Myelodysplastic syndromes	Busulfan-based	Bu, Flu, TT		1	3	
		TBI-based	TBI, Cy		1		
		Treo-based	Treo, Flu		1		
**Hemoglobinopathies**	Thalassemia	Treo-based	Treo, TT, Flu			5	6 (4.6%)
	Sickle cell disease	Treo-based	Treo, TT, Flu			1	
**Hemophagocytic lymphohistiocytosis (HLH)**		Busulfan-based	Bu, Cy, Eto			2	5 (3.8%)
		Other chemo-only	Flu, Mel			3	
**Inborn errors of metabolism**	Globoid leukodystrophy	Busulfan-based	Bu, Cy			1	4 (3.1%)
	X-linked adrenoleukodystrophy	Treo-based	Treo, TT, Flu			3	
**Primary Immunodeficiencies (PID)**	Wiskott-Aldrich syndrome	Busulfan-based	Bu, Cy, TT			1	2 (1.5%)
	X-linked lymohoproliferative	Busulfan-based	Bu, Cy			1	

Bu, busulfan; Cy, cyclophosphamide; Mel, melphalan; Eto, etoposide; Flu, fludarabine; TT, tiothepa; Treo, treosulfan.

Patients had been transplanted at a median age of 9.9 ± 4.9 years, while they were aged 21.4 ± 6.5 years upon the last endocrine evaluation. As an average, they had been followed-up in the setting of a dedicated long-term endocrine late-effects clinic for 9.0 ± 5.2 years.

**Figure 2:**
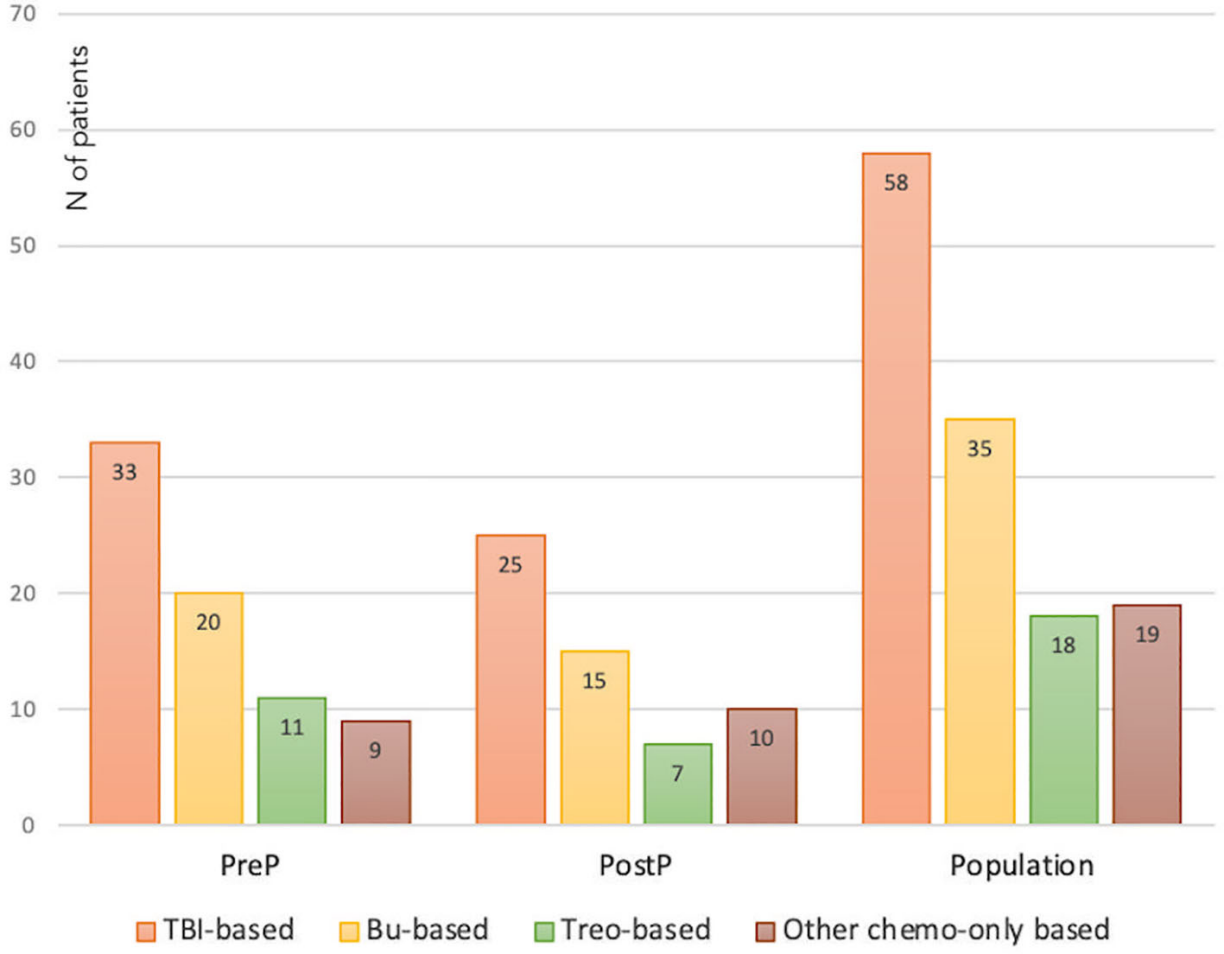
Distribution of the whole study population and of the PreP and PostP subcohorts with reference to the conditioning regimen administered before HSCT. TBI, total body irradiation; Bu, Busulfan; Treo, Treosulfan.

Seventy-three patients (56.2%) were prepubertal upon HSCT (PreP cohort), while 57 (43.8%) where either peri- or postpubertal (PostP cohort). Patients from the latter cohort were statistically older upon the last endocrine evaluation (21.2 ± 5.1 versus 17.0 ± 4.2 years, *p* 0.013). In **Figure 2**, patients within the whole study population and in each subcohort are distributed with reference to the conditioning administered before HSCT. In detail, 58 (44.6%) received a TBI-based, 35 (26.9%) a busulfan-based, 18 (13.8%) a treosulfan-based conditioning regimen and the remaining 19 (14.6%) a different chemo-only conditioning.

### 3.2 Clinical findings: pubertal progression and gonadal outcomes

The pubertal *status* recorded upon the last endocrine evaluation was consistent with Tanner stage 3, 4 and 5 in 19 (14.6%), 30 (23.1%) and 81 (62.3%) patients respectively, confirming that all the survivors enrolled were well established into puberty.

**Figure 3:**
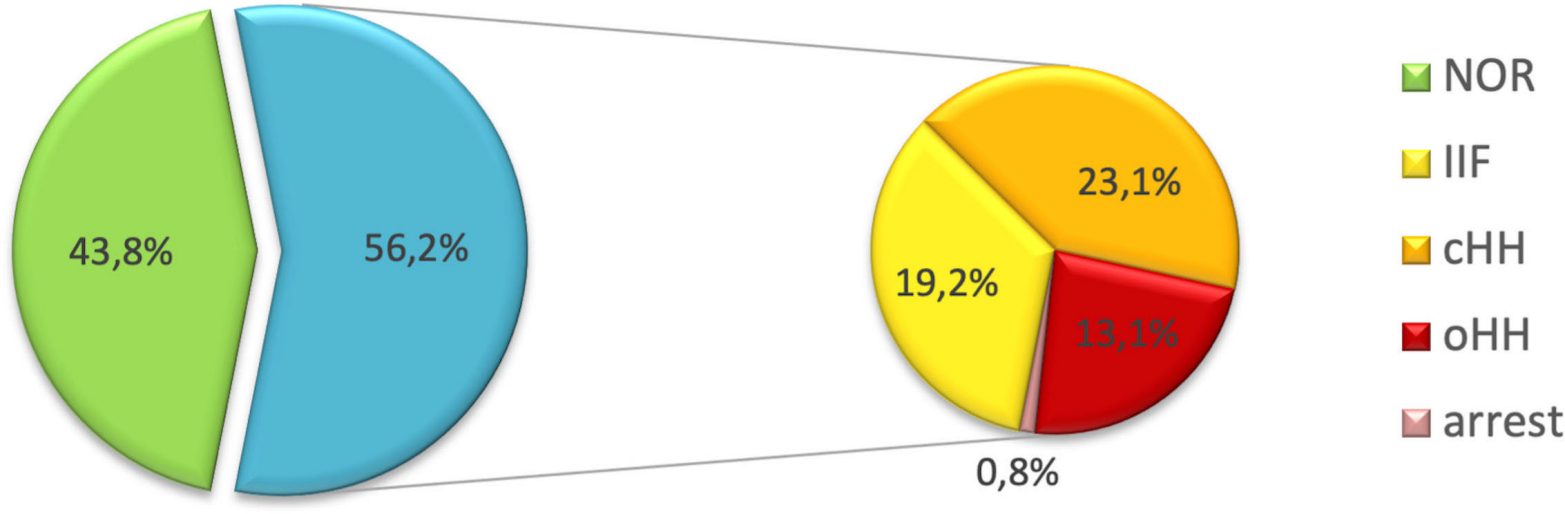
Distribution of gonadal outcomes in the study population. Overall, 43.8% of patients showed a spontaneous progression of puberty and a normal gonadal profile. On the other hand, 56.2% experienced one of the following clinical pictures: pubertal arrest (PA), isolated increase of FSH (IIF), compensated hypergonadotropic hypogonadism (cHH), overt hypergonadotropic hypogonadism (cHH).

Overall, 57 patients (43.8%) showed a spontaneous progression of puberty and normal LH, FSH and testosterone levels (NOR). The remaining 73 (56.2%) experienced either pubertal arrest (PA, n=1- 0.8%), isolated increase of FSH (IIF, n=25 – 19.2%), compensated hypergonadotropic hypogonadism (cHH, n=30 – 23.1%) or overt hypergonadotropic hypogonadism (cHH, n= 17 – 13.1%). **Figure 3** graphically represents the distribution of gonadal outcomes in the study population.

The patient diagnosed with PA showed no pubertal progression over more than 18 months (Tanner stage 3), progressive increase of LH and FSH and consensual decrease in testosterone levels, prompting the prescription of hormonal replacement therapy at the age of 14.8 years to induce complete pubertal attainment.

**Figure 4:**
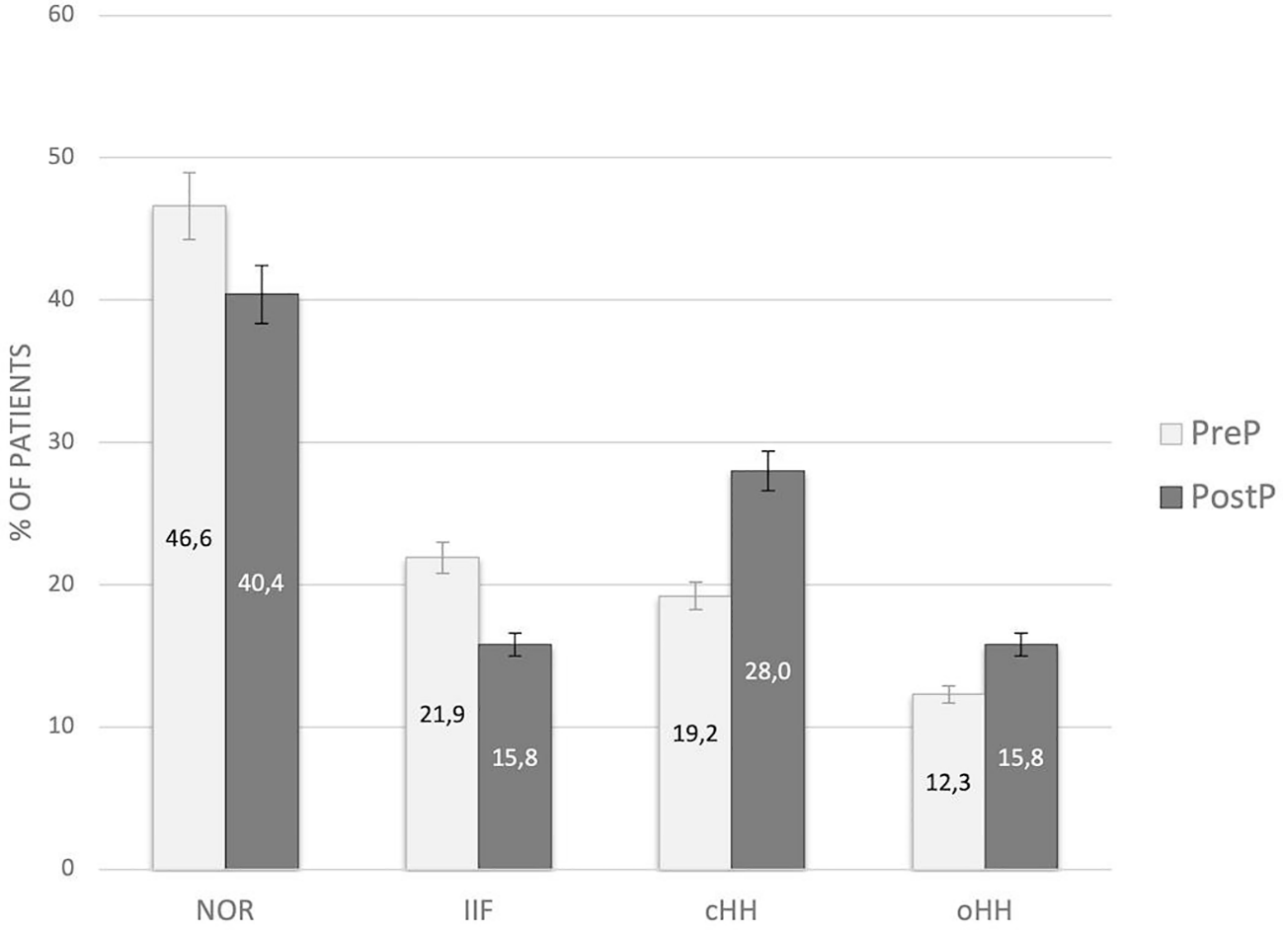
Distribution of gonadal outcomes in the study population with reference to *pubertal* status upon HSCT. PreP, patients prepubertal upon HSCT; PostP, patients peri-/postpubertal upon HSCT; NOR, normal gonadal function; IIF, isolated increase of FSH; cHH, compensated hypergonadotropic hypogonadism; oHH, overt hypergonadotropic hypogonadism.

No statistically significant difference in the distribution of gonadal outcomes was recorded between patients prepubertal and peri/post-pubertal upon transplantation (**Figure 4**).

### 3.3 Longitudinal assessment of clinical and biochemical data

**Figure 5:**
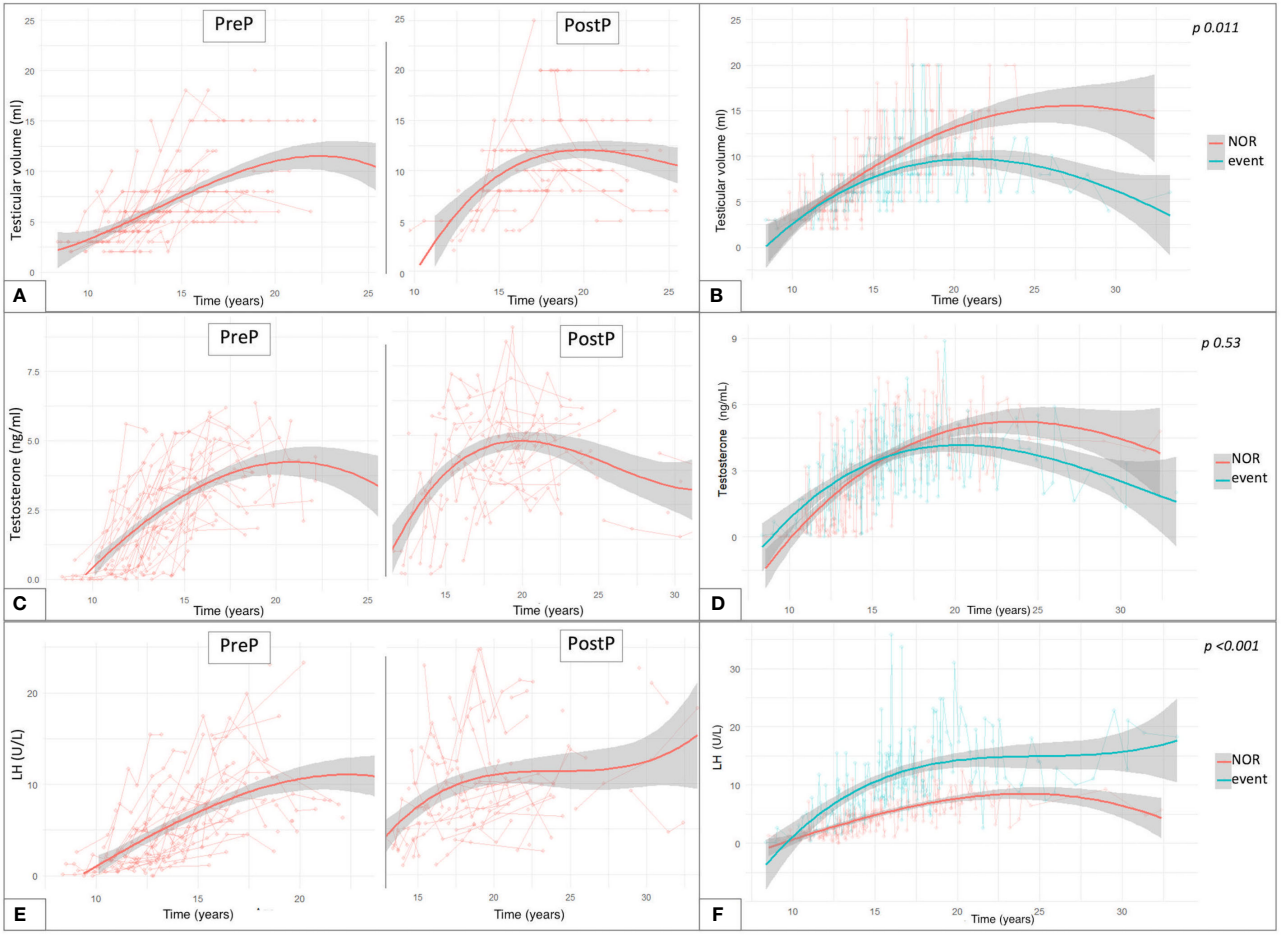
Trendlines of clinical and biochemical findings upon long-standing longitudinal endocrine evaluation plotted against patient’s age expressed in years. The longitudinal changes in testicular volume over time are reported with reference to the pubertal *status* upon HSCT **(A)** and to the gonadal outcome **(B)**. **(C)** reports the trendlines for testosterone levels in the PreP and PostP cohorts, while in **(D)** they are plotted based on the eventual development of gonadal impairment. Similar data are depicted for LH values recorded over time **(E, F)**. Overall, **(B, D, F)** compare the trendlines of testicular volume, testosterone levels and LH values between patients with normal endocrine results versus those who experienced gonadal impairment (event). By “event” we defined any degree of gonadal damage recorded in the study population (isolated increase in FSH, compensated and overt hypogonadism).

By longitudinally assessing testicular volume (TV) over time, we compared the trends of testicular growth among patients from the PreP versus the PostP cohorts. The latter showed average larger adult TV, compared to patients transplanted before pubertal onset, as depicted in **Figure 5A**. Out of 81 patients who had achieved Tanner stage 5, TV was statistically greater in the PostP (12.2 ± 5.1 ml) than in the PreP cohort (10.3 ± 4.1 ml, *p* 0.049).

**Figure 5B** reports the interpolation curves of the TV recorded longitudinally among patients who did not experience clinical or biochemical findings consistent with testicular damage (NOR) versus those who developed any endocrine disfunction (IFF, cHH or oHH). The trend lines show a progressive gap from 12 years onwards, with the latter presenting with smaller TV and with the longitudinal model showing statistical significance (*p* 0.011).

Concerning testosterone levels, we depicted the values longitudinally recorded in the study population over time (**Figure 5C**). Out of the 81 patients who had spontaneously achieved Tanner stage 5, no statistically significant differences were found in the last testosterone values recorded (before any eventual hormonal replacement therapy was undertaken) in the PreP (4.13 ± 1.81 ng/ml) versus the PostP (4.53 ± 1.91 ng/ml) cohorts (*p* 0.34). In addition, no statistically significant differences were recorded in the trendlines between hypogonadal versus event-free patients (*p* 0.53, panel D).

Finally, as showed in panel E, LH and testosterone levels showed a specular trend between 20 and 30 years, when a progressive decrease in sexual steroids was associated with a compensatory increase in luteinizing hormone. Overall, statistically superimposable adult LH levels were drawn among fully post-pubertal patients from the PreP (10.6 ± 7.71 U/L) and PostP (11.15 ± 6.27 U/L) cohorts (*p* 0.728). Consistently, no differences were recorded in the distribution of FSH levels between the two subcohorts (PreP: 25.54 ± 20.62 versus PostP: 21.45 ± 15.84, *p* 0.318).

### 3.4 The impact of conditioning regimens on gonadal outcomes

**Figure 6:**
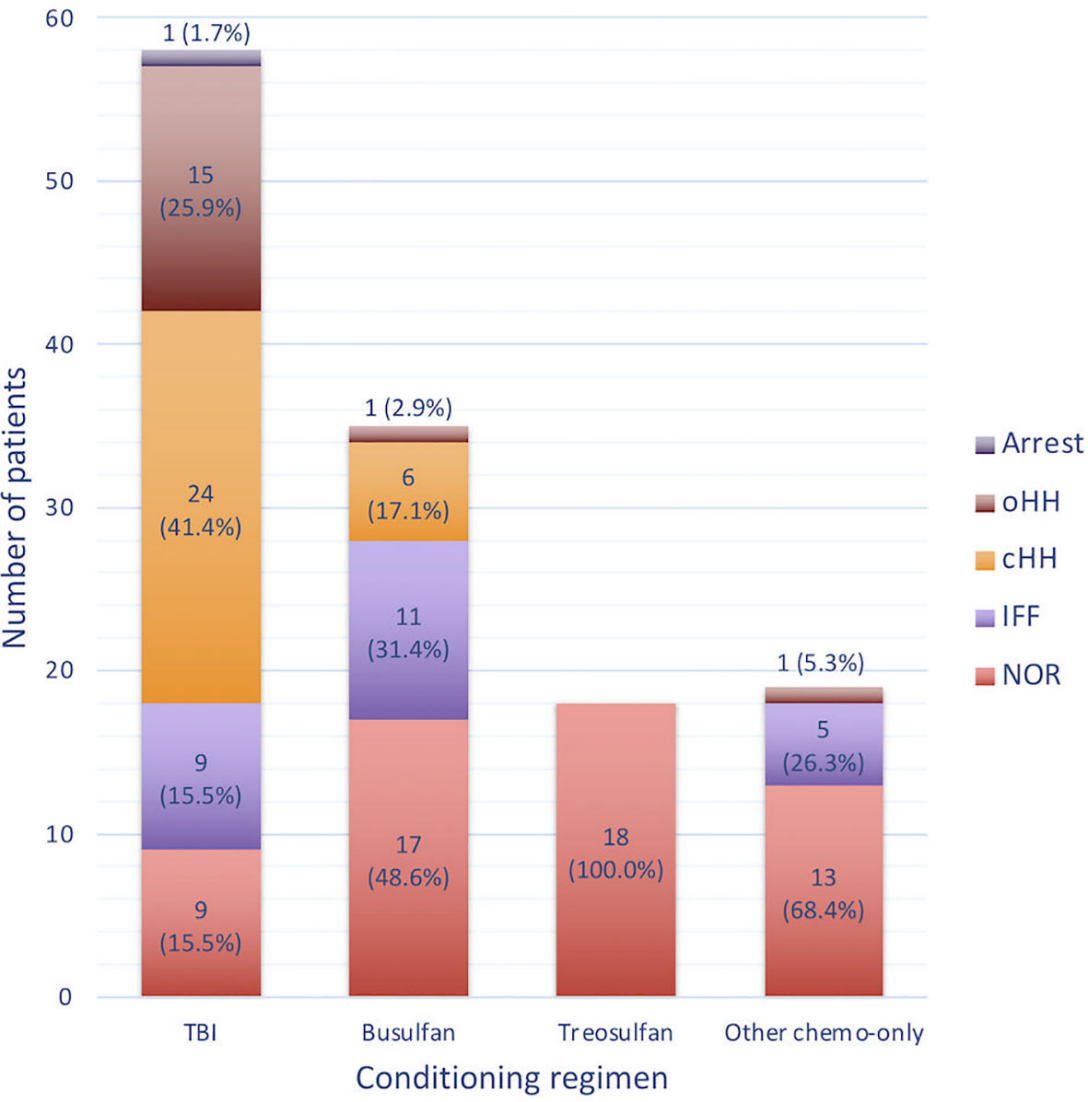
Histogram representing the occurrence of the different gonadal outcomes with reference to the conditioning regimen class administered before transplantation. NOR, normal gonadal function; IIF, isolated increase of FSH; cHH, compensated hypergonadotropic hypogonadism; oHH, overt hypergonadotropic hypogonadism.

**Figure 6** reports the distribution of gonadal outcomes based on the conditioning received. Overall, a certain degree of gonadal dysfunction (ranging from isolated increase of FSH to hypogonadism or pubertal arrest) was recorded in 84.5% of the patients following TBI, 51.4% after a busulfan-based conditioning and 31.6% after cyclophoshamide/fludarabine, whereas no abnormal findings were found among the 18 patients exposed to treosulfan.

**Figure 7:**
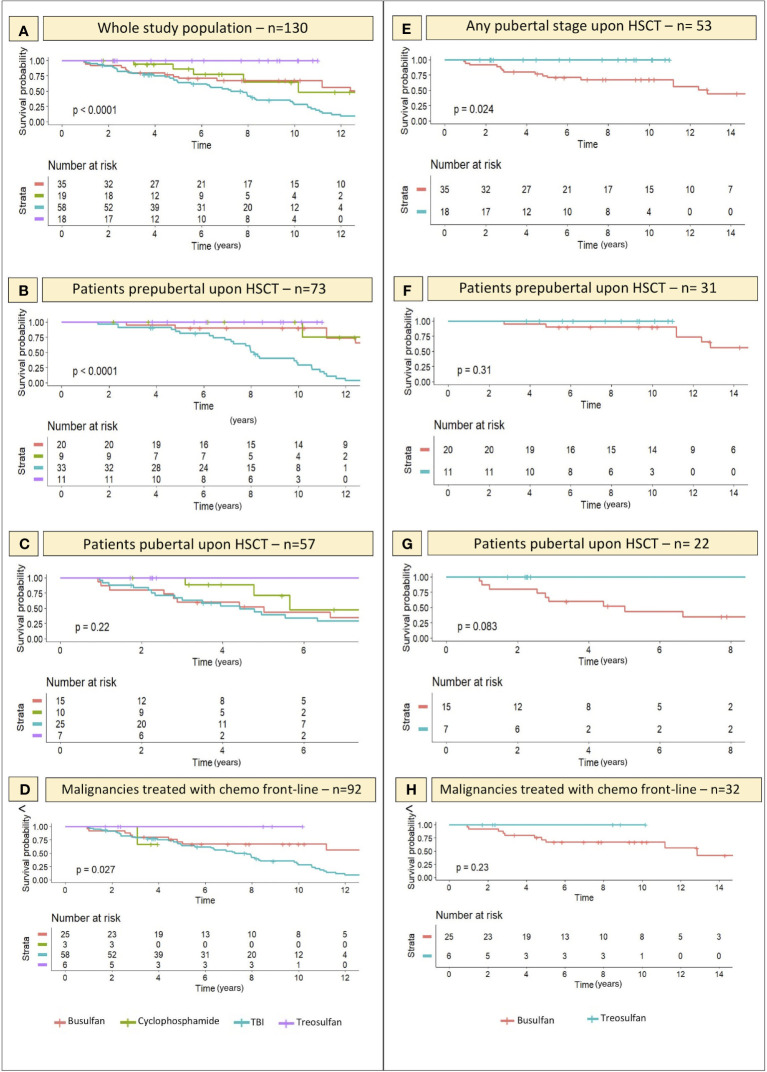
Kaplan-Meier curves representing the likelihood (y axis) of an event-free course over time with reference to the conditioning schedule administered before transplantation. On x axis the years elapsed following HSCT are reported. **(A–C)** depict the gonadal function survival probability for all the 4 conditioning classes assessed within the whole study population and in the PreP and PostP cohorts, respectively. **(E)** specifically focus on the comparison of the gonadal function survival probability in patients conditioned with treosulfan- versus busulfan-based regimens and shows that the difference is statistically significant. **(F, G)** report the same comparison in the PreP and PostP cohorts, respectively. Finally, **(D, H)** show the likelihood of an event-free course in the selected cohort of patients diagnosed with malignancies and exposed to chemotherapy before the conditioning regimen, as a front-line treatment. Statistical significance set for *p*<0.05.

The event-free survival probability (**Figure 7**) of the four subcohorts treated with different conditioning regimens (TBI, busulfan, treosulfan and cyclophosphamide) was evaluated for the whole study population irrespectively of baseline pubertal *status* (Panel A), for patients prepubertal upon HSCT (Panel B) and for those for whom puberty had already kicked-in at the time of transplantation (Panel C).

By “event” we defined any degree of gonadal damage recorded (IIF, cHH or oHH).

Though not significant in the PostP cohort (*p* 0.22), a remarkable statistical difference was recorded in the distribution of events among patients exposed to different conditioning regimens before the onset of puberty (*p <*0.0001) and in the whole study population (*p <*0.0001). Total body irradiation and busulfan showed the most impaired gonadal function survival curves, while the survival probability was as high as 100% among the 18 patients conditioned with treosulfan-based schedules.

When selectively comparing the occurrence of gonadal events recorded in patients conditioned with busulfan versus those exposed to treosulfan, we found a statistically significant difference in the survival curves of the two cohorts (*p* 0.024), with busulfan showing a remarkably greater gonadotoxicity (**Figure 7E**). Similar trends, though not statistically significant, were recorded when splitting the study population into the PreP and PostP subcohorts.

**Figure 8:**
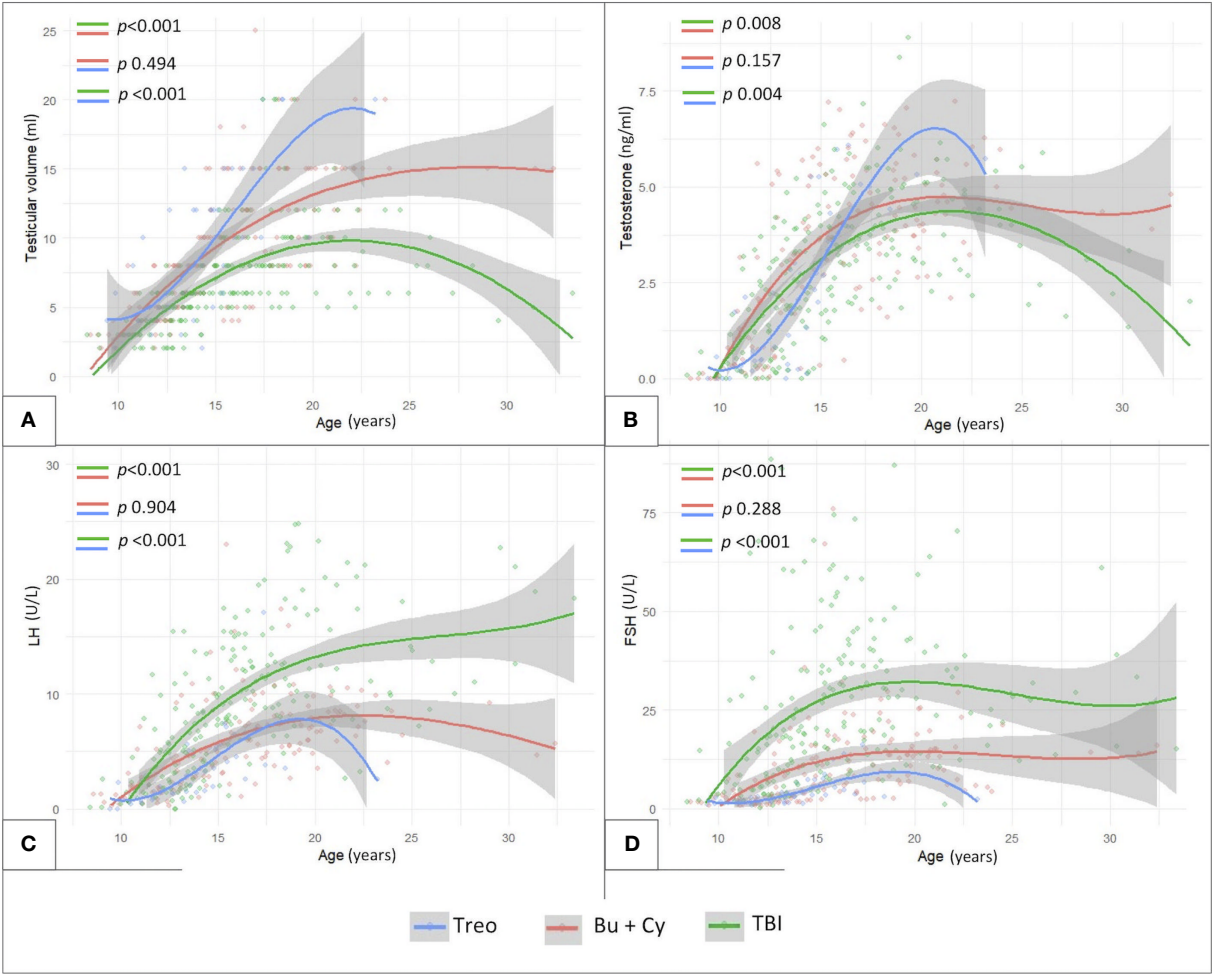
Longitudinal trend of testicular volume **(A)**, testosterone **(B)**, LH **(C)** and FSH levels **(D)** plotted against patients’age with reference to three conditioning classes assessed: TBI-, treosulfan- and busulfan- based. treo, treosulfan; bu, busulfan; cy, cyclophosphamide; TBI, total body irradiation. Statistical significance set for *p*<0.05.

Finally, **Figure 8** shows the longitudinal trends of testicular volume (panel A), testosterone (panel B), LH (panel C) and FSH (panel D) levels over time with reference to the conditioning regimen administered before HSCT. Busulfan/cyclophosphamide-based conditioning regimens were associated with statistically larger median testicular volumes (*p <*0.001), higher testosterone levels (*p* 0.008) and lower LH/FSH levels (*p <*0.001) than TBI. On the other hand, patients from the abovementioned Bu-Cy cohort were found to present with smaller testicular volumes, lower testosterone and higher LH/FSH levels than following the administration of treosulfan, though the difference was not statistically different (**Figure 8**, *p* 0.494, *p* 0.157, *p* 0.904 and *p* 0.288, respectively).

### 3.5 The impact of alkylating agents’ cumulative dose on gonadal outcomes

In order to assess gonadal damage with reference to the burden of alkylating agents received, we firstly excluded patients exposed to TBI, as the detrimental synergetic role of radio- and chemotherapy cannot be quantified and CED alone would result in a severe underestimation of the iatrogenic effects of antineoplastic treatments on testicular function.

**Figure 9:**
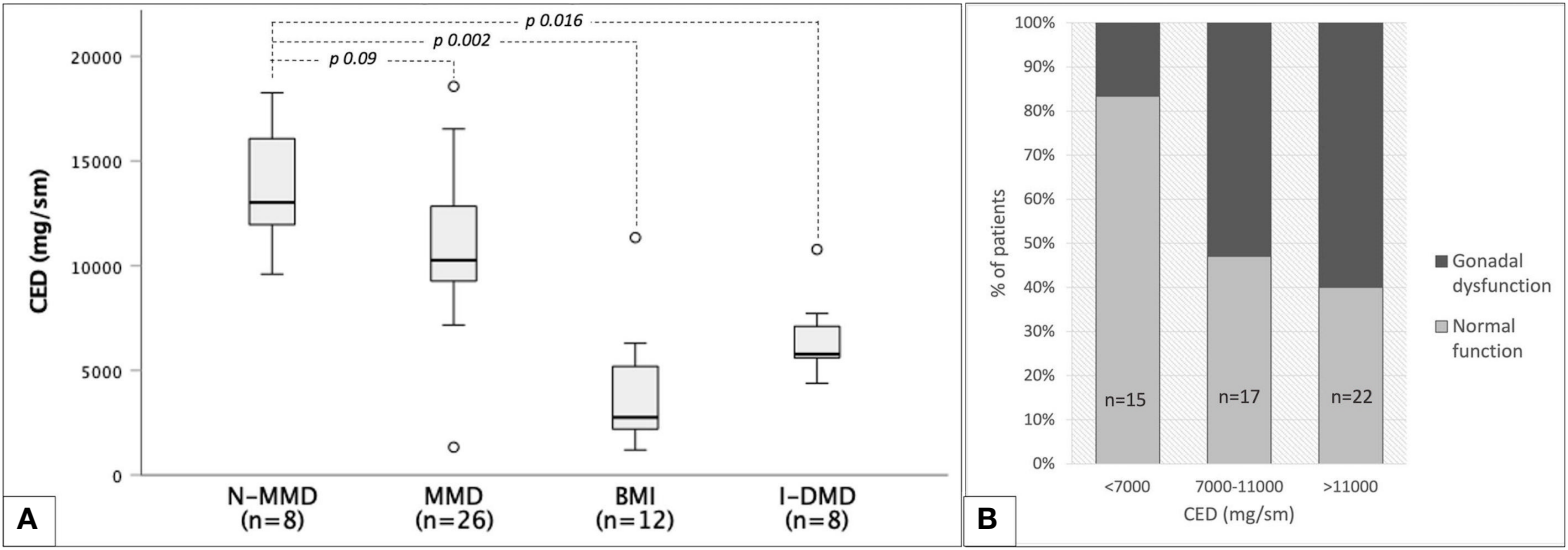
Cyclophosphamide equivalent dose (CED) and gonadal function among 54 TBI- and treosulfan-free patients. **(A)** box plots representing median CED values with reference to classes of baseline diseases: non-myeloid malignant disorders (N-MMD), myeloid malignant disorders (MMD), bone marrow insufficiencies (BMI), inborn dysimmune or metabolic disorders (I-DMD). **(B)** occurrence of any degree of gonadal dysfunction in three progressively increasing CED classes.

Out of 72 non-irradiated patients, CED could be estimated among 54 men exposed to chemo-only treosulfan-free regimens. As showed in **Figure 9A**, CED mean values were superimposable among myeloid and TBI-free non-myeloid malignant disorders (10,9 ± 3,5 versus 13,7 ± 2,8 g/m^2^, *p* 0.09), while they were statistically lower in non-oncological diseases (6.5 ± 1.9 in patients transplanted for inborn error of metabolism or immunity and 3.9 ± 2.8 g/m^2^ in bone marrow insufficiency disorders).

CED values showed no statistically significant correlation with testosterone (r=-0.091, *p* 0.58) or LH values (r=0.161, *p* 0.28). A statistically significant though unsatisfactory positive correlation was found only between CED and FSH levels (r=0.42, *p* 0.04). **Figure 9B** shows the progressive increase in the occurrence of gonadal dysfunction along with CED, with the two variables being statistically associated (*p* 0.017, Fisher’s exact test).

### 3.6 The role of front-line chemotherapy on gonadal outcomes

In order to describe the detrimental role of alkylating agents eventually administered before the conditioning regimen, we also assessed gonadal outcomes in a selected subcohort of patients diagnosed with malignant disorders and treated with chemotherapy front-line (front-line chemotherapy cohort, FLCC). The FLCC included 92 patients diagnosed with acute leukemias or lymphomas.

**Figure 10:**
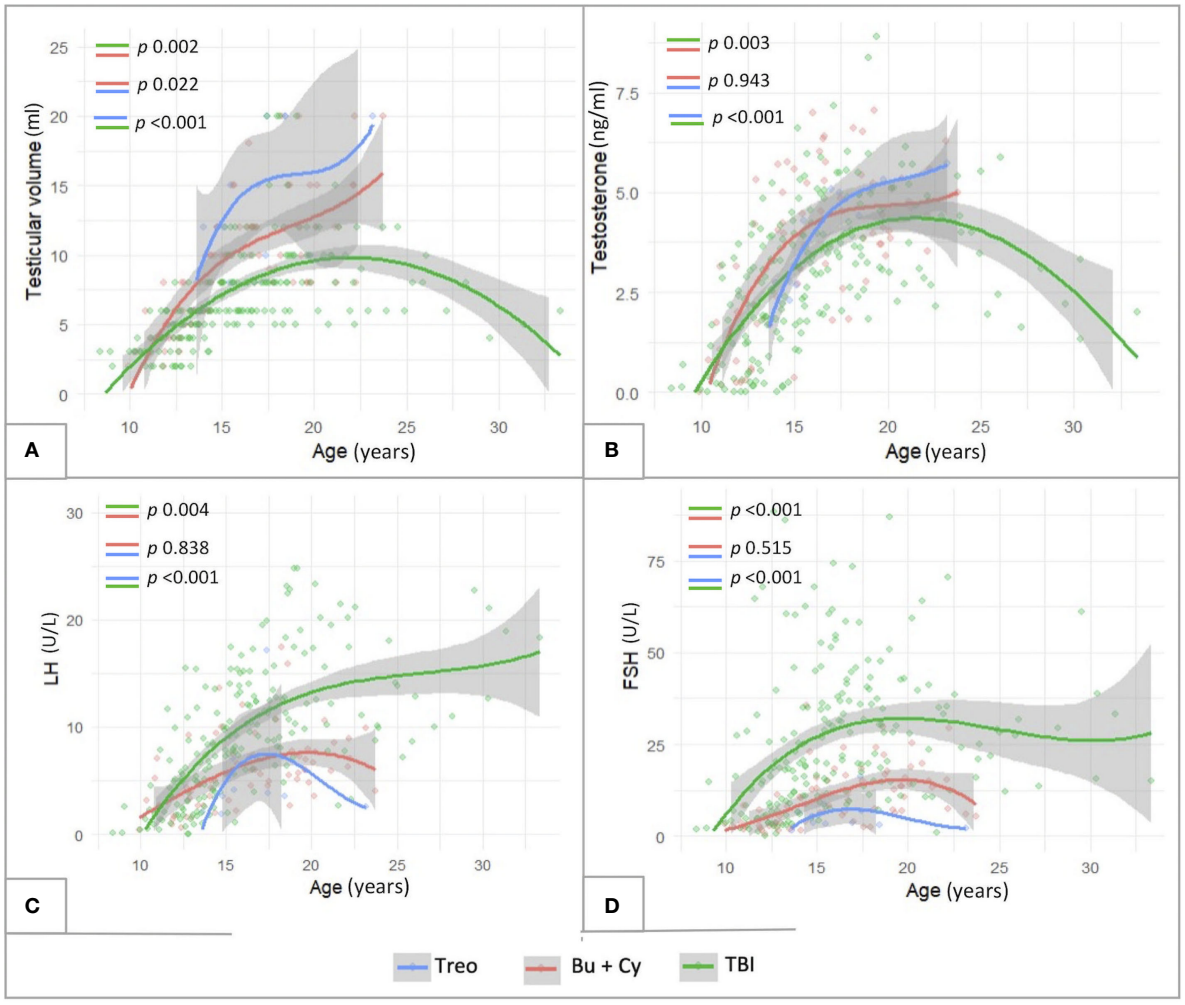
Longitudinal trend of testicular volume **(A)**, testosterone **(B)**, LH **(C)** and FSH levels **(D)** plotted against patients’age within the subcohort of patients diagnosed with malignancies and exposed to chemotherapy front-line (FLCC, front-line chemotherapy cohort). The trendlines are reported with reference to three conditioning classes assessed: TBI-, treosulfan- and busulfan- based. treo, treosulfan; bu, busulfan; cy, cyclophosphamide; TBI, total body irradiation. Statistical significance set for *p*<0.05.

As showed in **Figure 10**, also in this selected subcohort, as consistent with the general population, TBI-free patients achieved statistically greater TV and testosterone levels (*p*<0.01) and lower LH/FSH levels (*p*<0.01) compared to irradiated ones. In addition, the comparison between Bu-Cy- and Treo-conditioned patients confirmed also in the FLCC the results drawn in the whole population, with trendlines towards greater testosterone levels and lower LH/FSH values among the latter. Furthermore, the longitudinal assessment of TV over time showed statistically greater TV following treosulfan versus Bu-Cy regimens (*p* 0.022).

Finally, Kaplan-Maier curves confirmed a statistically different biological behavior of gonadal function over time among patients conditioned with Treosulfan versus Busulfan versus TBI (*p* 0.027) in the FLCC (**Figures 7D, H**).

### 3.7 Determinants of gonadal outcomes following HSCT: multivariable analysis

By applying a Cox multivariable regression model, we assessed the integrated contribution of the following variables on gonadal outcomes: conditioning regimen (busulfan, treosulfan, cyclophosphamide, TBI), background disease (malignant disorder requiring chemotherapy versus other disorders), chronic GvHD (score 0-1 versus 2 or greater), age upon the last biochemical evaluation and pubertal stage at HSCT (PreP versus PostP). Overall, conditioning regimen and pubertal status upon HSCT were the only variables showing a statistically significant impact on testicular outcomes. In detail, exposure to TBI was associated with a 2-fold increase in the risk of gonadal failure compared to busulfan (HR 2.34, CI 1.08-8.40, p 0.047). On the other hand, being pre-pubertal upon HSCT could be regarded as a protective factor, as it was associated to a remarkably reduced risk of developing any degree of testicular damage (HR 0.15, CI 0.08-0.30, p <001).

## 4 Discussion

Endocrine disorders represent an important share of the late effects experienced by patients undergoing HSCT in pediatric age and young adulthood and account for up to 70% of the adverse events recorded years or decades following transplantation (8). In addition, impaired gonadal function can be regarded as the most frequent endocrine *sequela* among transplant survivors (19). Indeed, both alkylating agents and irradiation severely affect testicular and ovarian function, potentially leading to both infertility and endocrine insufficiency.

Despite the abovementioned remarkable burden of gonadal dysfunction among HSCT survivors, the challenges in providing a systematic endocrine assessment of pubertal attainment over time and the lack of universally shared protocols for monitoring and treating hypogonadism in this selected population has resulted in an overall limited amount of published analyses systematically focusing on the longitudinal course of clinical and biochemical findings concerning testicular function following transplantation.

In order to detail the long-term trend of clinical and biochemical markers of testicular function over time, we performed a retrospective longitudinal analysis of data recorded over a 30-year endocrinological follow-up in a tertiary care HSCT unit. All the patients enrolled had undergone a 6- to 12-monthly endocrine assessment, as a part of a dedicated follow-up focused on endocrine disorders.

Overall, the detection of clinical/biochemical signs consistent with impaired Leydig cell function raised from 36.9% in the study population up to 84.5% in the TBI cohort. These findings are consistent with the data published by other authors (11, 20).

Germ cells are remarkably more sensitive to chemotherapy and radiotherapy than testosterone-secreting Leydig cells. Radiation doses as low as 2–6 Gy, busulfan >600 mg/kg and cyclophosphamide >7.5–9 g/m^2^ are the threshold above which spermatogonial cell depletion and subsequent oligo-azoospermia occur (9, 21). On the other hand, impairment in testosterone secretion occurs following more gonadotoxic treatment protocols. Accordingly, 19.2% of our patients experienced an isolated increase of FSH, indirect biochemical sign consistent with germ cell damage, despite normal LH and testosterone levels.

Upon a long-standing longitudinal follow-up, we found that patients who underwent HSCT prior to puberty achieved statistically smaller TV than those who underwent HSCT after the onset of pubertal maturation. When assessing patients who had achieved Tanner stage 5, statistically smaller median TV was found in patients who were prepubertal at the time of HSCT. These results, consistent with those reported by Wilhelmsson and colleagues and de Kloet and colleagues (20, 22), seem to collide with the remarkable reduction of the risk of developing hypogonadism among patients prepubertal upon HSCT versus those peri-/post-puberal, as this supports the hypothesis of a protective role of younger age and prepubertal status on gonadal health following transplantation. This apparent paradox can be easily explained considering that most patients from the PostP cohort had already achieved a certain TV before undergoing transplantation. As TV does not necessarily shrink following gonadotoxic treatments, this results in an underestimation of the severity and occurrence of testicular impairment, making TV an unreliable marker of endocrine dysfunction in patients transplanted after the onset of puberty. In addition, as about 90% of TV is represented by seminiferous tubules and as germ cells are remarkably more chemo- and radio-resistant than testosterone-secreting ones, no systematic correlation between TV and Leydig cell function can be drawn among childhood cancer survivors. Indeed, suboptimal TV is commonly found among transplanted patients showing retained testosterone levels and who had achieved complete and persistent virilization throughout puberty and afterwards. In this setting, we would expect that markers of germ cell function (i.e. inhibin B) would represent a promising biochemical indicator of residual fertility, though wide-scale studies need to be set to exploit its reliability among transplanted patients.

Nevertheless, by performing a longitudinal clinical assessment following HSCT, we highlighted that the gap in the TV between patients who developed hypogonadism and those with a normal endocrine function appeared to progressively widen over time, suggesting a compromised testicular maturation in hypogonadal patients, especially among those still prepubertal upon HSCT.

By longitudinally assessing patients’ endocrine function over time, we outlined that testosterone levels show a downwards trend and, consistently, we recorded a compensatory progressive increase in LH levels. Our data suggest that transplantation is followed by a progressive deterioration of Leydig cells function as years elapse after HSCT. As already postulated by published guidelines about the follow-up of childhood cancer survivors (23), these findings emphasize the need for life-long follow-up of gonadal health in patients who underwent HSCT, as a remarkable reduction of testosterone may occur when increased LH values no longer compensate a progressively declining testicular function.

As already widely reported (24), our results highlight a remarkably greater occurrence of either compensated or overt hypogonadism following TBI than after radiation-free regimens. In this setting, the novelty of our data stands in the longitudinal assessment of clinical (TV) and biochemical parameters of testicular function over time, that led us to highlight a statistically different biological behavior following TBI versus chemo-only regimens. In our study population, the gap in TV, testosterone levels, LH and FSH between irradiated and non-irradiated patients became progressively more overt as years elapsed following HSCT, supporting the hypothesis of a time-dependent detrimental effect of radiation on testicular function.

Consistently with these data, irrespectively of the conditioning regimen, onset of puberty occurred spontaneously in all the patients enrolled, while only 1 out of 58 irradiated boys experienced PA. The remarkable percentage of post-pubertal occurrence of hypogonadism in spite of a preserved onset and progression of early pubertal attainment supports the hypothesis of a slowly progressing TBI-induced deterioration of Leydig cell function over time.

Concerning chemo-only conditioning regimens, though a trend towards a worse gonadotoxicity profile of busulfan compared to treosulfan had already been described in both genders, to the best of our knowledge our analysis is the first that provides a statistically significant demonstration that the latter is associated, in males, to a remarkably lower cumulative incidence of hypogonadism upon a long-standing longitudinal assessment. As displayed by Kaplan-Meier curves, while none of the 18 patients exposed to treosulfan-based conditionings experienced any biochemical signs of testicular dysfunction, a progressively increasing percentage of patients conditioned with busulfan was diagnosed with hypogonadism over time, with this difference being more evident for patients post-pubertal upon HSCT.

In a cornerstone study conducted in 2019 by Faraci et al. on behalf of the European Bone Marrow Transplantation Society, treosulfan was associated to statistically higher rates of spontaneous puberty and menarche in girls, while a similar but non-statistically significant trend was recorded in males conditioned with busulfan (n=47) versus treosulfan (n=6) (14).

Consistently, in 2020 Leiper and colleagues demonstrated on a wide population of patients exposed to reduced-intensity conditioning scheduled for non-malignant disorders that treosulfan was associated to statistically higher inhibin B levels than busulfan-cyclophosphamide and fludarabine-melphalan regimens (25). Our results support the thesis of a gonadal-sparing profile of treosulfan, but shed light on a slightly different focus. On the one hand, we specifically reported endocrine function disorders rather than markers of residual fertility. In addition, our study population mostly received more gonadotoxic myeloablative doses of alkylating agents for either malignant or non-malignant disorders rather than reduced intensity schedules.

Out of 134 male patients exposed to HSCT, de Kloet et al. found 46% of gonadal dysfunction following busulfan compared to 14% in patients with treosulfan-based conditioning, but the difference was not statistically significant (15). On a superimposable sample, we managed to demonstrate a statistically significant gap in the cumulative incidence of gonadal adverse endocrine outcomes after treosulfan versus busulfan mostly thanks to the remarkable amount of biochemical and clinical data recorded across a three-decade longitudinal assessment of pubertal attainment, that probably represents the main strength of our analysis. Consistently, in the subcohort of patients diagnosed with malignant disorders, we also demonstrated that patients conditioned with treosulfan achieved statistically greater testicular volumes compared to busulfan. Nevertheless, the difference in the trendlines of testosterone and LH/FSH levels over time, though consistent with a lower degree of gonadal damage after treosulfan, did not achieve statistical significance.

The use of treosulfan in the setting of HSCT conditioning is relatively recent. Accordingly, the average follow-up among treo-conditioned patients was shorter than following busulfan in our study population (6.5 ± 3.4 versus 10.7 ± 6.1, *p*<0.01). The differences in the biological behavior of gonadal function in the two cohorts is strikingly overt upon the Kaplan-Meier curves, with the longitudinal survivorship model being statistically significant and with gonadal events starting to occur soon after busulfan administration (**Figure 7**). Nevertheless, it cannot be excluded that treosulfan is associated to a later-onset gonadal dysfunction compared to busulfan. Accordingly, only an extension of the follow-up period would confirm the hypothesis of a long-lasting gonadal-sparing profile of treosulfan compared to other alkylating agents.

Acknowledging the inherent constraints of our research, we must address certain limitations. As most patients enrolled are cancer survivors, it is reasonable to believe that antineoplastic therapies administered before transplantation may have played a synergic role with HSCT-related gonadal toxicity. On the other hand, even though current chelating regimens are widely applied and effective, patients transplanted for hemoglobinopathies may suffer from a variable degree of iron overload-related gonadal damage. Theoretically, a selected population of patients transplanted for the same hematological disorder and exposed to identical pre-HSCT treatments would represent the gold standard in assessing late effects among survivors. As HSCT represents the ultimate treatment solution in a small percentage of patients, a multicentric assessment would be required to achieve a sufficient sample fulfilling these criteria. Nevertheless, the lack of shared follow-up programs among non-oncologic transplanted patients would lead to a remarkable inter-center variability, thus resulting in a poor reproducibility of the results achieved.

Indeed, the application of a strict follow-up protocol for all the patients enrolled represents one of the strengths of our monocentric analysis. In addition, none of the 7 patients transplanted for malignant disorders and exposed to antineoplastic treatments before HSCT developed hypogonadism following treosulfan conditioning.

Secondly, we are aware that assessing the specific detrimental role of a single alkylating agent administered as a part of a polychemotherapy protocol may result in misleading conclusions. In detail, most patients from the busulfan subcohort received both busulfan and cyclophosphamide, while those from the treosulfan subcohort were mostly conditioned with treosulfan *plus* either tiothepa and fludarabine or fludarabine and cyclophosphamide. Nevertheless, the occurrence of hypogonadism following treosulfan *plus* cyclophosphamide was as low as 0%, while combining cyclophosphamide *plus* busulfan resulted in a remarkable gonadotoxicity.

Thirdly, given its retrospective nature, some data could not be entirely retrieved and were therefore missing. Accordingly, though the study is focused on Leydig cell residual function, additional markers of germ cell function would have provided interesting additional information about testicular health following HSCT. Nevertheless, given the long-standing three-decade longitudinal assessment, retrospectively gathering direct (semen analysis) or indirect (inhibin B values) indexes of fertility was not feasible.

Lastly, a certain degree of inter-operator variability could be reported when assessing testicular volume and Tanner staging over a long-lasting longitudinal evaluation. Nevertheless, over three decades, only four endocrinologists (all with commitment and expertise in the field of cancer survivorship) have been involved in the systematic evaluation of transplanted patients in our Institution.

## 5 Conclusions

Firstly, by reporting the longitudinal trendlines of clinical and biochemical markers of testicular function over a dedicated three-decade endocrine follow-up, we demonstrated that patients prepubertal upon transplantation were exposed to a reduced risk of developing hypogonadism following HSCT, despite overall smaller testicular volume achieved in adulthood.

Secondly, we showed the downwards trend in testosterone levels after the achievement of full spontaneous pubertal attainment, compensated by an inverse upwards trend in LH levels, as consistently with a progressively declining Leydig cell function over time.

Finally, our data confirmed also in male patients the gonadal-sparing profile of treosulfan compared to busulfan-based regimens, with a statistically lower occurrence of hypogonadism and a trend towards larger testicular volume, higher testosterone levels and lower gonadotropins upon a systematical longitudinal assessment.

## Data availability statement

The raw data supporting the conclusions of this article will be made available by the authors, without undue reservation.

## Ethics statement

The studies involving humans were approved by Comitato Etico della Brianza - Monza e Brianza. The studies were conducted in accordance with the local legislation and institutional requirements. Written informed consent for participation was not required from the participants or the participants’ legal guardians/next of kin in accordance with the national legislation and institutional requirements.

## Author contributions

AC: Conceptualization, Investigation, Supervision, Validation, Writing – original draft, Writing – review & editing. MN: Data curation, Writing – original draft, Writing – review & editing. GC: Data curation, Formal analysis, Investigation, Supervision, Writing – review & editing. AGad: Data curation, Formal analysis, Investigation, Writing – review & editing. SM: Data curation, Investigation, Validation, Writing – review & editing. SL: Data curation, Writing – review & editing. ABuo: Data curation, Writing – review & editing. SO: Data curation, Writing – review & editing. GS: Data curation, Writing – review & editing. IB: Data curation, Writing – review & editing. FV: Data curation, Supervision, Writing – review & editing. GO: Data curation, Supervision, Writing – review & editing. AGai: Supervision, Writing – review & editing. GF: Supervision, Writing – review & editing. ABio: Supervision, Validation, Writing – review & editing. ABal: Data curation, Supervision, Validation, Writing – original draft, Writing – review & editing.
